# Excretion of dengue virus RNA by *Aedes aegypti* allows non-destructive monitoring of viral dissemination in individual mosquitoes

**DOI:** 10.1038/srep24885

**Published:** 2016-04-27

**Authors:** Albin Fontaine, Davy Jiolle, Isabelle Moltini-Conclois, Sebastian Lequime, Louis Lambrechts

**Affiliations:** 1 Insect-Virus Interactions Group, Department of Genomes and Genetics, Institut Pasteur, Paris, France; 2 Equipe Résidente de Recherche d’Infectiologie Tropicale, Division Expertise, Institut de Recherche Biomédicale des Armées, Brétigny-sur-Orge, France; 3 Centre National de la Recherche Scientifique, Unité de Recherche Associée 3012, Paris, France; 4 Université Pierre et Marie Curie, Cellule Pasteur UPMC, Paris, France

## Abstract

Successful transmission of a vector-borne pathogen relies on a complex life cycle in the arthropod vector that requires initial infection of the digestive tract followed by systemic viral dissemination. The time interval between acquisition and subsequent transmission of the pathogen, called the extrinsic incubation period, is one of the most influential parameters of vector-borne pathogen transmission. However, the dynamic nature of this process is often ignored because vector competence assays are sacrificial and rely on end-point measurements. Here, we report that individual *Aedes aegypti* mosquitoes release large amounts of dengue virus (DENV) RNA in their excreta that can be non-sacrificially detected over time following oral virus exposure. Further, we demonstrate that detection of DENV RNA in excreta from individual mosquitoes is correlated to systemic viral dissemination with high specificity (0.9–1) albeit moderate sensitivity (0.64–0.89). Finally, we illustrate the potential of our finding to detect biological differences in the dynamics of DENV dissemination in a proof-of-concept experiment. Individual measurements of the time required for systemic viral dissemination, a prerequisite for transmission, will be valuable to monitor the dynamics of DENV vector competence, to carry out quantitative genetics studies, and to evaluate the risk of DENV transmission in field settings.

Vector competence is the intrinsic ability of arthropods to acquire and subsequently transmit vector-borne pathogens, such as, malaria parasites or dengue viruses (DENV)^1^. Experimental vector competence assessments of arthropod populations are an important component of assessing the risk of vector-borne disease. Vector competence is a quantitative trait that varies not only between arthropod species, but also within a vector species. For example, 24 populations of the mosquito *Aedes aegypti* sampled throughout Mexico and the United States displayed substantial variation in their vector competence for DENV^2^.

Evaluating vector competence experimentally for human pathogens often cannot be ethically carried out because it requires measuring actual transmission from the vector to a naïve human host. Alternative methods rely on proxies such as the use of animal models. For dengue, existing animal models such as non-human primates and genetically modified mice are ethically sensitive and costly^3^, therefore many investigators use DENV detection in mosquito saliva collected *in vitro* as evidence of virus transmission potential^4^. However, the small amount of virus that can be recovered by this method often requires enhancing sensitivity by subsequent virus amplification such as intrathoracic inoculation of recipient mosquitoes^5^,^6^,^7^. Moreover, the presence and amount of saliva collected may vary between individual mosquitoes and is difficult to assess, which reduces the accuracy of the method. Alternatively, detection of DENV infectious particles or RNA in mosquito head or leg tissues has been used as a convenient proxy for transmission potential^8^,^9^,^10^. Infectious titer measured in mosquito heads or legs correlates well with the probability to detect infectious virus in saliva samples collected *in vitro*^11^. Testing heads in particular allows standardizing the amount of tissue tested across mosquitoes and its technical simplicity facilitates the processing of larger numbers of samples. Importantly, all DENV transmission assays described above require sacrificing the mosquito, which prevents sequential measurements on the same individual at multiple, subsequent time points.

Successful DENV transmission by a mosquito will occur if the virus overcomes at least two different anatomical ‘barriers’ that can prevent a systemic infection^12^. The ‘midgut infection barrier’ prevents the virus from establishing infection in the midgut epithelium following an infectious blood meal. The ‘midgut escape barrier’ blocks systemic dissemination of virus from the midgut cells to the hemocoel, secondary target tissues and thus infection of the salivary glands. The molecular mechanisms underlying these barriers have not been elucidated. The time interval between virus acquisition during blood feeding to eventual transmission to another host is referred to as the extrinsic incubation period (EIP). EIP is one among the most influential parameters for transmission of vector-borne pathogens^13^. The sooner the virus is transmitted, the greater the probability of successful transmission events before the vector dies. Mean EIP for DENV is in the range of 10–15 days at 25 °C^14^.

Whereas infection status of a mosquito (i.e., whether a midgut infection is persistently established or not) is determined early after virus exposure, the overall potential for DENV transmission in a population of infected mosquitoes is expected to increase exponentially over time. This translates into a sigmoid function of the cumulative proportion of transmitting mosquitoes over time^15^,^16^. Because of the dynamic nature of transmission potential, vector competence is usually scored in laboratory studies based on the proportion of infectious mosquitoes at a single, late time point after imbibing an infectious blood meal. This approach ignores variation in the rate of vector competence change over time and can be misleading in comparisons of transmission potential^15^. End-point measurements of vector competence provide an estimate of maximum vector competence, but cannot be used to infer EIP.

Here, we report the discovery that females of the DENV vector *Ae. aegypti* with a disseminated DENV infection excrete large amounts of viral RNA that can be detected non-sacrificially from individual mosquitoes. This discovery supports a practical method for monitoring variation in the timing of DENV dissemination, and thus potential for virus transmission, across multiple individual mosquitoes. The method relies on detection of viral RNA in mosquito excreta collected passively. The non-destructive nature of this method offers a unique opportunity to study the dynamic process of vector-virus interactions by measuring the time required for viral dissemination (i.e., a proxy for EIP) in single mosquitoes.

## Results

### Mosquitoes can excrete large amounts of viral RNA following oral exposure to DENV

**Figure 1:**
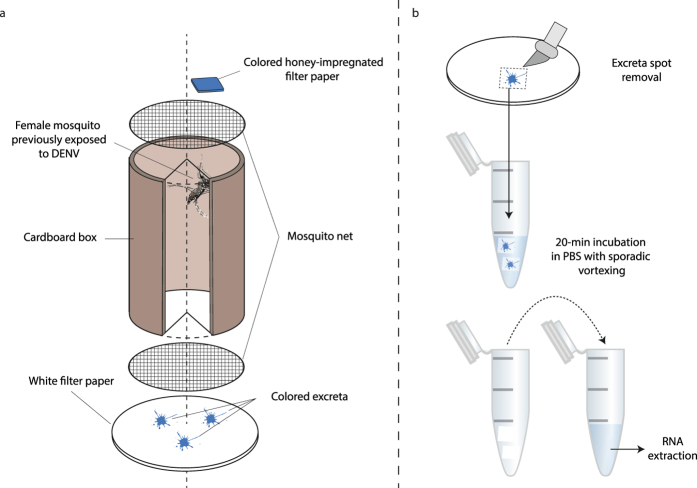
Schematic of the method used to collect mosquito excreta in time series. (**a**) Following oral exposure to a DENV infectious blood meal, individual female mosquitoes were maintained in cardboard boxes (80-mm high and 44 mm in diameter) and continuously exposed to a 1-cm^2^ square of colored honey-impregnated filter paper. (**b**) At each sampling time point, pieces of filter paper placed below the cardboard boxes were visually inspected for colored excreta spots that were excised with a scalpel. Excised excreta spots were soaked in PBS for 20 min and viral RNA was extracted from the solution.

Our approach is based on the detection of viral RNA in individual mosquito excreta in time series. The method relies on the observation that mosquitoes produce colored excreta when they are maintained with access *ad libitum* to a honey solution mixed with a food colorant. Excreta spots are easily visualized on a white piece of filter paper placed below a cardboard box in which the mosquitoes are maintained, and can be collected for subsequent testing (Fig. 1). Mosquitoes do not produce colored excreta in the absence of dye in the honey solution. Based on a detection threshold of 10 DENV RNA copies/μL of sample by reverse transcription and quantitative polymerase chain reaction (RT-qPCR) and an elution volume of 75 μL, we estimated that at least 1.5 × 10^3^ RNA copies (equivalent to 3.18 log_10_ units) had to be present in the filter paper to be subsequently detected in our assay.

**Figure 2:**
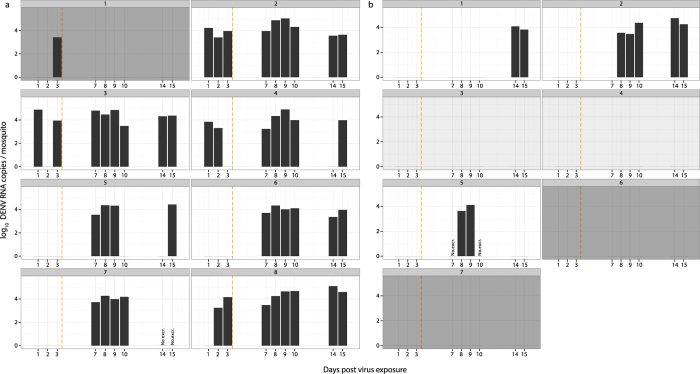
Quantity of DENV RNA detected in excreta from individual mosquitoes following virus exposure. Each panel represents the log_10_-transformed number of DENV RNA copies detected in excreta from individual *Ae. aegypti* females (numbered above the panels). In (**a**) mosquitoes were exposed to a high virus dose (2.14 × 10^7 ^FFU/mL), whereas in (**b**) they were exposed to a low virus dose (1.36 × 10^5 ^FFU/mL). Detection of DENV RNA directly from the blood meal is unlikely to have occurred after the end of blood digestion, indicated by a vertical orange dashed line. Dark grey backgrounds represent mosquitoes that did not become infected (i.e., with a midgut infection barrier). Light grey backgrounds represent mosquitoes that became infected but that did not develop a disseminated infection (i.e., with a midgut escape barrier). White backgrounds represent mosquitoes that developed a disseminated infection. ‘No. excr.’ indicates the lack of visible excreta on the filter paper.

In a first experiment, female *Ae. aegypti* were offered blood meals containing either a high dose (2.14 × 10^7^ focus-forming units [FFU]/mL) or a low dose (1.36 × 10^5^ FFU/mL) of DENV. A total of 18 fully engorged females were placed individually in cardboard boxes as described in Fig. 1a. The presence of DENV RNA in excreta was monitored on days 1, 2, 3, 7, 8, 9, 10, 14 and 15 post exposure. At day 15, the final midgut infection and viral dissemination status of each mosquito was determined based on the presence of DENV RNA in the carcass and head tissues, respectively. Three mosquitoes (one exposed to the high virus dose and two exposed to the low virus dose) died before day 15 and were excluded from further analysis. With very few exceptions, colored excreta were observed every day, which allowed the presence of excreted DENV RNA to be assessed on a daily basis during the EIP (Fig. 2). Dark excreta spots resulting from blood digestion could be observed at the bottom of the cardboard boxes during days 1–3 post blood feeding. Blood is typically no longer visible in dissected midguts beyond day 3 after a DENV infectious blood meal (unpublished observation). Therefore, we assumed that detection of DENV RNA in excreta on day 7 or later was unlikely to result from contamination with viral RNA contained in digested blood.

Out of eight mosquitoes exposed to the high virus dose, seven became infected and developed a disseminated infection. Out of seven mosquitoes exposed to the low virus dose, two did not become infected, two became infected but did not develop a disseminated infection, and three developed a disseminated infection. Considering only positive samples across virus doses, the total amount of DENV RNA detected in excreta ranged from 1.71 × 10^3^ to 1.24 × 10^5^ RNA copies per female. The amount of viral RNA detected in positive samples did not differ statistically between doses (Wilcoxon rank sum test, *p* = 0.568). DENV RNA was detected before day 3 post exposure in the excreta of five out of eight mosquitoes (62.5%) exposed to the high virus dose (Fig. 2a). Conversely, before day 3 post exposure DENV RNA remained undetectable in the excreta of all mosquitoes exposed to the low virus dose (Fig. 2b). DENV RNA was detected in excreta between day 7 and day 15 post exposure for all mosquitoes exposed to the high virus dose except for female #1 that did not become infected (Fig. 2a). Among mosquitoes exposed to the low virus dose, DENV RNA was detected between day 8 and day 15 post exposure but only for the three mosquitoes that developed a disseminated infection (Fig. 2b).

In mosquitoes that had a confirmed disseminated infection, DENV RNA was almost continuously detected in excreta. Only in a few instances, no DENV RNA was detected at individual time points despite the presence of colored excreta spots on the filter paper, probably reflecting amounts of viral RNA below the detection threshold. Importantly, in mosquitoes that did not become infected (i.e., with a midgut infection barrier) DENV RNA was never detected after day 3 post exposure regardless of the virus dose in the blood meal. In addition, DENV RNA was not detected in two mosquitoes exposed to a low virus dose that became infected but did not develop a disseminated infection (i.e., with a midgut escape barrier). This result is consistent with the notion that excretion of DENV RNA could be associated with systemic viral dissemination.

### Presence of DENV RNA in mosquito excreta is strongly correlated to viral dissemination

To assess whether the presence of DENV RNA in mosquito excreta is associated with midgut infection and/or viral dissemination status, 33 *Ae. aegypti* females were orally exposed to either 10^7^ FFU/mL, 4 × 10^5 ^FFU/mL or 10^5 ^FFU/mL of DENV in a second experiment. Fully engorged females were collected at days 5, 8 and 15 post exposure to cover a range of vector competence phenotypes. Their head, midgut and Malpighian tubules were freshly dissected and subsequently tested separately for DENV RNA. The excreta of each female were also tested for DENV RNA just prior to collection. Females were assigned to three vector competence categories: uninfected, infected without a disseminated infection, and infected with a disseminated infection. Infection status was based on detection of DENV RNA in the midgut, whereas dissemination status relied on detection of DENV RNA in head tissues and/or Malpighian tubules.

Among the 33 females collected, 10 (30.3%) were uninfected, 9 (27.3%) were infected without a disseminated infection and 14 (42.4%) were infected with a disseminated infection. Only one out of 14 mosquitoes with a disseminated viral infection had a DENV negative head and DENV positive Malpighian tubules. Presence of DENV RNA in the head was strongly correlated with presence of DENV RNA in the Malpighian tubules (rho = 0.99, *p* = 1.5 × 10^−7^). Presence of DENV RNA in the head and in the Malpighian tubules was also associated, to a lesser extent, with presence of DENV RNA in the midgut (rho = 0.80, *p* = 1.3 × 10^−2^ and rho = 0.83, *p* = 8.5 × 10^−3^, respectively) (Table 1).

A total of 10 females (30%) had DENV RNA positive excreta. Presence of DENV RNA in excreta was not significantly associated with midgut infection. Only 10 out of 23 midgut-infected females (43%) had DENV RNA positive excreta. However, presence of DENV RNA in excreta was significantly associated with presence of DENV RNA in the head tissues and in the Malpighian tubules (rho = 0.90, *p* = 9.5 × 10^−4^ and rho = 0.87, *p* = 2.5 × 10^−3^, respectively) (Table 1). Nine of the 10 females (90%) with DENV RNA detected in their excreta had a disseminated DENV infection.

Specificity of a pathogen diagnostic test refers to the probability that a test result is negative when the pathogen is not present (i.e., true negative rate). Sensitivity refers to the probability that a test result is positive when the pathogen is present (i.e., true positive rate)^17^. Mosquitoes with a confirmed disseminated infection (i.e., detection of DENV RNA in head tissues or Malpighian tubules) that released DENV RNA in their excreta were considered as true positives (TP). Mosquitoes with DENV RNA in their excreta but without a confirmed disseminated infection were considered as false positives (FP). Mosquitoes without DENV RNA in their excreta and without a confirmed disseminated infection were considered as true negatives (TN). Finally, mosquitoes with a confirmed disseminated infection but with no DENV RNA detected in their excreta were considered as false negatives (FN). Presence of DENV RNA in mosquito excreta used as a proxy for viral dissemination is highly specific (TN/(FP+TN) = 18/(18+1) = 0.95), but moderately sensitive (TP/(FN+TP) = 9/(5+9) = 0.64). It is worth noting that the single FN female with positive Malpighian tubules but negative excreta had a negative head. Because it was exposed to a low dose (4 × 10^5 ^FFU/mL) of DENV and sampled at day 8 post infection it is likely that viral dissemination had just begun. Sensitivity increases to 0.69 when dissemination is determined by the presence of DENV RNA in head tissues regardless of the infection status of Malpighian tubules.

In addition to the filter paper used to collect excreta at the bottom of the cardboard boxes, honey-impregnated filter paper squares used as the sugar feeding substrate (Fig. 1) were also tested for the presence of DENV RNA for 15 of the 33 mosquitoes in the second experiment. Presence of colored excreta on the filter paper placed below the cardboard boxes was interpreted as evidence of colored honey intake, and thus salivation on the filter paper square. Sensitivity and specificity of DENV RNA detection in excreta and honey-impregnated filter paper squares as a proxy for viral dissemination was calculated for this subset of samples. A total of 9 out of 15 mosquitoes (60%) had a confirmed disseminated DENV infection, determined by the presence of DENV RNA in both head tissues and Malpighian tubules. DENV RNA was detected in 8 excreta samples and only 3 honey-impregnated filter paper squares. Both methods had a specificity of 1 but the sensitivity of DENV RNA detection in excreta was higher (0.89) than in honey-impregnated filter paper squares (0.33). The mean log_10_-transformed quantity (±standard deviation) of DENV RNA in honey-impregnated filter papers (3.45 ± 0.33) was significantly lower (Wilcoxon rank sum test, *p* = 0.028) than in excreta (4.36 ± 0.71).

### Monitoring DENV RNA in excreta from individual mosquitoes can reveal biological differences in viral dissemination dynamics

A third experiment was conducted to evaluate whether the presence of DENV RNA in mosquito excreta could be used to detect differences in the time required for systemic viral dissemination (i.e., a proxy for EIP) in individual mosquitoes. The experiment was designed to measure differences in time to dissemination between mosquitoes initially exposed to a different virus dose. A total of 24 *Ae. aegypti* females were orally exposed to either 10^7 ^FFU/mL (high virus dose) or 4.5 × 10^5 ^FFU/mL (low virus dose) of DENV and monitored daily for the presence of DENV RNA in their excreta. Three mosquitoes (one in the high virus dose and two in the low virus dose) were excluded from further analysis because they did not produce colored excreta over a prolonged period after blood feeding. Visual absence of colored excreta spots could result from lack of sugar feeding, lack of excretion, excretion of colorless excreta, or excretion on concealed surfaces such as the walls of the cardboard box. Two thirds of the mosquitoes (66.7%) died during the course of the experiment, but survival was not significantly different between virus doses (likelihood ratio test, *p* = 0.895). The experiment was terminated at day 17 post exposure for the high virus dose and at day 27 post exposure for the low virus dose, respectively. We assumed that most dissemination events would have occurred at day 17 post exposure for the high virus dose, which we retrospectively verified.

**Figure 3:**
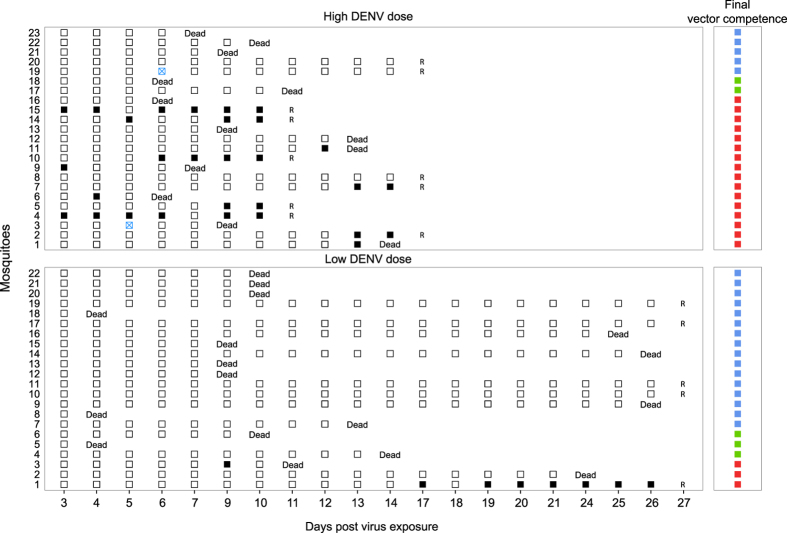
Time series of DENV RNA detection in excreta from individual mosquitoes following virus exposure. Each row corresponds to an individual mosquito, numbered on the left side of the chart. The main panels show the presence of DENV RNA in excreta as a function of day post virus exposure during the lifetime of each individual mosquito. In the upper part of the chart mosquitoes were exposed to a high virus dose (10^7 ^FFU/mL), whereas in the lower part mosquitoes were exposed to a low virus dose (4.5 × 10^5 ^FFU/mL). Solid black squares represent excreta positive for DENV RNA, and hollow squares represent excreta negative for DENV RNA. Hollow blue squares with a cross indicate the lack of visible excreta on the filter paper. A letter R corresponds to the time at which mosquitoes were removed from the experiment. The smaller panels on the right side of the chart show the final vector competence status of each mosquito (blue squares: uninfected; green squares: midgut-infected without dissemination; red squares: infected with a disseminated viral infection).

Following their death or removal at the end of the experiment, females were assigned to three final vector competence categories: uninfected, infected without a disseminated infection, and infected with a disseminated infection. Midgut infection and viral dissemination status were determined based on detection of DENV RNA in the carcass and in head tissues, respectively. A significantly lower proportion of mosquitoes became infected when they were exposed to the low virus dose (6/22 = 27%) compared to the high virus dose (18/23 = 78%). Likewise, a significantly lower proportion of midgut-infected mosquitoes developed a disseminated infection with the low virus dose (3/6 = 50%) than with the high virus dose (16/18 = 89%). Across virus doses, DENV RNA could be detected in the excreta from 13 out of 19 mosquitoes (68%) with a disseminated DENV infection. Importantly, no DENV RNA was detected in excreta from mosquitoes that did not become infected or mosquitoes that became infected but did not develop a disseminated infection (Fig. 3), corroborating the previous results. Based on the definitions provided above, the sensitivity and specificity of the method were 0.68 and 1, respectively.

The time until first detection of DENV RNA in excreta from individual mosquitoes was compared between the high and low virus doses using a Cox regression model. The estimated hazard (±standard error) of viral dissemination for the high virus dose was 12.8 ± 1.07 times higher than for the low virus dose. The virus dose was significantly positively associated with the probability of DENV RNA detection in excreta (likelihood ratio test, *p* = 0.00151) and thus negatively associated with the length of time to dissemination (Supplementary Figure 1). Using DENV RNA detection in excreta as a proxy for viral dissemination, the mean number of days for viral dissemination to occur (±standard deviation) was 7.75 ± 4.22 days for the high virus dose and 13 ± 5.66 days for the low virus dose.

## Discussion

We described a novel, non-sacrificial method that exploits mosquito sugar feeding to detect DENV RNA in excreta collected in time series from individual *Ae. aegypti* females. Importantly, we showed that the presence of DENV RNA in mosquito excreta was correlated to systemic viral dissemination with high specificity and moderate sensitivity. The underlying mechanism remains to be determined, but the strong association between viral dissemination and DENV RNA excretion could result from infection of the mosquito excretory system (i.e., Malpighian tubules) and/or disposal of degradation products from other infected tissues. Delayed detection of DENV antigens in Malpighian tubules relative to other organs including the midgut^18^ favors the latter hypothesis. In any case, it is unlikely that Malpighian tubules can become infected directly from the midgut lumen without prior viral dissemination to the hemocoel. Reciprocally, an earlier study found that the proportion of mosquitoes with a disseminated DENV infection was temporally decoupled from the amount of DENV RNA detected in the midgut^19^, indicating that DENV RNA excretion cannot be used to infer viral burden in the midgut.

Our experiments only used one *Ae. aegypti* population exposed to a single DENV isolate, therefore additional work is required to determine whether viral RNA excretion by mosquitoes is a general phenomenon. The presence of a flavivirus in mosquito excreta was reported in 1929 by Aragaõ and Da Costa Lima who examined fecal material from infectious *Ae. aegypti* mosquitoes^20^. In their study, mosquito excreta contained infectious yellow fever virus from day 5 after the infectious blood meal^20^,^21^, consistent with a correlation between virus excretion and systemic infection. In the present study, whether infectious DENV particles can be recovered in mosquito excreta using our method was not investigated. The number of infectious DENV particles can be 2 to 5 log_10_ units lower than viral RNA copies^22^, making detection of infectious DENV particles considerably less sensitive in low concentration samples.

A disseminated viral infection is necessary but not sufficient for DENV transmission^12^,^13^. Therefore, our method based on detection of DENV RNA in excreta will not replace existing transmission assays. However, it is consistent with use of a new experimental strategy to study the dynamics of vector competence in individual mosquitoes over the course of their lifetime. Ye and colleagues recently developed a non-destructive assay to repeatedly quantify DENV RNA in *Ae. aegypti* saliva from groups of 10 mosquitoes^23^. This method also exploits mosquito sugar feeding but is based on detection of DENV RNA in a microtube cap containing a sucrose solution and placed upside down on the bottom of the cup where the mosquitoes are housed. Although a saliva-based method is most relevant to evaluate transmission potential, this experimental setup does not completely avoid fecal contamination, which may result in false positives^23^. In our experimental design, the average amount of DENV RNA detected in mosquito excreta was significantly higher than in the sugar substrate. Pooling mosquitoes in the saliva-based assay may be required to compensate for the fact that individual mosquitoes release very small amounts of DENV RNA their saliva.

A major asset of our method is the fact that it provides a measure at the level of individual mosquitoes. Until now, individual variation in DENV dissemination or EIP could not be studied because of the sacrificial nature of conventional vector competence assays. One significant advantage of individually assigning a value to each mosquito is the greater flexibility in the models that can be used to analyze time series. In previous experimental studies that explicitly examined variation in the duration of DENV EIP in *Ae. aegypti*, EIP was scored using different batches of mosquitoes collected at different time points following virus exposure^14^,^16^,^24^. This strategy does not provide a single EIP value for each mosquito, but rather measures the cumulative proportion of transmitting mosquitoes at each sampling interval. Such data can be analyzed with a logistic regression of the proportion of transmitting mosquitoes as a function of time post virus exposure. However, logistic regression implies that 100% of the mosquitoes are expected to be transmitting at some time post virus exposure, an assumption that is not always verified. In most vector competence studies a proportion of mosquitoes never transmit virus regardless of the duration of EIP e.g.,^7^,^25^.

Time to event analyses, also known as survival analyses, are well adapted to analyze data produced from follow-up studies in which the outcome variable is the time until the occurrence of an event of interest^26^. Unlike conventional regression methods, time to event models can also account for missing events over the course of the study (i.e., censoring). In our experiments, censoring was useful to include mosquitoes that died prior to observing viral dissemination. In order to maximize intake from the honey-impregnated substrate, we did not provide mosquitoes with a source of water, which may have reduced their survival. Time to event models could help to better parameterize equations of vectorial capacity^27^,^28^,^29^, which describe the complex interplay of factors that influence the dynamics of vector-borne pathogen transmission.

An additional advantage of individually scoring the time required for DENV dissemination is the large body of quantitative genetic methods that can be employed to study the genetic basis of this trait^30^. For example, quantitative trait locus (QTL) mapping is a powerful method based on individual phenotypes. It consists of locating chromosomal regions that affect a quantitative trait (i.e., QTL) by measuring genotype-phenotype associations across a genetic map^31^. Another classical approach to study the genetic basis of a quantitative trait is to artificially select for extreme phenotypes^30^,^32^. Identifying *Ae. aegypti* females with a disseminated viral infection non-destructively will significantly facilitate experiments designed to select for DENV resistance or susceptibility. Based on the presence of DENV RNA in their excreta, females previously exposed to an infectious blood meal would be easily sorted according to their vector competence status and allowed to lay eggs separately after exposure to a non-infectious blood meal. This process can be repeated iteratively during several mosquito generations to obtain lines with contrasted dissemination phenotypes and assess their response to selection.

Finally, detection of DENV RNA in mosquito excreta, coupled with mosquito traps, may contribute to refine tools to monitor the risk of DENV transmission in field conditions. The potential of mosquito sugar feeding behavior for arbovirus surveillance was previously demonstrated with a system based on detection of viral RNA from honey-soaked nucleic acid preservation cards combined with a mosquito trap^33^. The original study showed that viral RNA was preserved up to seven days on the card, so that the trap could be used continuously to monitor virus presence in vector populations without the need to handle or process the insects^33^. Although until now it was assumed that trapped mosquitoes expectorated virus on the card, in the light of the present study we suggest that some of the viral RNA detected could have been excreted. Nevertheless, combining detection of viral RNA in both saliva and excreta should be explored as a way to enhance the sensitivity of this promising method. In our study, detecting DENV RNA in excreta spots was more sensitive than in the sugar substrate.

In conclusion, we showed that DENV RNA can be continuously detected over time in excreta from individual *Ae. aegypti* females with a disseminated viral infection. This finding has several practical implications for vector competence studies both in the field and in the laboratory. Non-sacrificial monitoring of DENV dissemination allows each individual mosquito to be assigned a single time interval for viral dissemination, which offers unique opportunities to study the quantitative genetic basis of DENV vector competence dynamics. It could also strengthen the arsenal of surveillance tools that can be deployed in field conditions to monitor the risk of DENV transmission.

## Methods

### Ethics

The Institut Pasteur animal facility has received accreditation from the French Ministry of Agriculture to perform experiments on live animals in compliance with the French and European regulations on care and protection of laboratory animals. This study was approved by the Institutional Animal Care and Use Committee at Institut Pasteur.

### Virus

A wild-type, low-passage DENV-1 isolate (KDH0026A) was used to challenge mosquitoes experimentally. This isolate that was originally recovered from the serum of a clinically ill dengue patient attending Kamphaeng Phet Provincial Hospital, Thailand as previously described^31^. Informed consent of the patient was not necessary because the virus isolated in laboratory cell culture was no longer considered a human sample. The isolate was passaged three times in *Aedes albopictus* C6/36 cells prior to its use in this study. The full-length consensus genome sequence of the isolate is available from GenBank under accession number HG316481. To prepare virus stock, sub confluent C6/36 cells were infected with 0.2 mL of inoculum in a 25-cm^2^ culture flask and incubated for 7 days at 28 °C with 5 mL of Leibovitz’s L-15 medium supplemented with 0.1% penicillin (10,000 U/mL)/streptomycin (10,000 μg/mL) (Life Technologies, Grand Island, NY, USA), 1x non-essential amino acids (Life Technologies) and 2% fetal bovine serum (FBS, Life Technologies). At the end of the incubation, the cell culture medium was harvested, adjusted to 10% FBS and pH ~8 with sodium bicarbonate, aliquoted and stored at −80 °C.

Virus titration was performed by standard focus-forming assay (FFA) modified from a published protocol^34^. This assay relies on inoculation of 10-fold dilutions of a sample onto a sub confluent culture of C6/36 cells, followed by incubation and subsequent visualization of infectious foci by indirect immunofluorescence. Briefly, C6/36 cells were seeded in a 96-well plate and grown overnight. Serial dilutions were prepared in Leibovitz’s L-15 medium supplemented with 0.1% penicillin [10,000 U/mL] / streptomycin [10,000 μg/mL], 1x non-essential amino acids and 2% FBS. The culture medium was removed and cells were inoculated with 40 μL/well. After 1 hour of incubation at 28 °C, the inoculum was removed and cells were overlaid with 150 μL/well of a 1:1 mix of overlay medium (Leibovitz’s L-15 medium, 0.1% penicillin [10,000 U/mL]/streptomycin [10,000 μg/mL], 1x non-essential amino acids, 2x Antibiotic-Antimycotic (Life Technologies), and 10% FBS) and 3.2% carboxyl methylcellulose solution, and incubated for 5 days at 28 °C. Cells were fixed by addition of 150 μL/well of 3.6% formaldehyde in phosphate-buffered saline (PBS) and incubation at room temperature (20–25 °C) for 20 min. The overlay and fixative were removed and the cells were washed three times with PBS, followed by 30 min permeabilization with 50 μL/well of 0.3% Triton X-100 (Sigma-Aldrich, St Louis, MO, USA) in PBS at room temperature. The cells were washed three times in PBS and incubated for 1 hour at 37 °C with 40 μL/well of mouse anti-dengue virus complex monoclonal antibody MAB8705 (Merck Millipore, Molsheim, France) diluted 1:200 in PBS + 1% bovine serum albumin (BSA) (Interchim, Montluçon, France). After another three washes in PBS, cells were incubated at 37 °C for 30 min with 40 μL/well of an Alexa Fluor 488-conjugated goat anti-mouse antibody (Life Technologies) diluted 1:500 in PBS + 1% BSA. After three more washes in PBS and a final wash in ultrapure water, infectious foci were counted under a fluorescent microscope and converted into focus-forming units/mL (FFU/mL).

### Mosquitoes

Experiments were carried out with adult *Ae. aegypti* mosquitoes derived from a field population originally sampled in the Muang District of Kamphaeng Phet Province, Thailand. Experiments took place within 10 generations of laboratory colonization. Mosquitoes were maintained under controlled insectary conditions (28 ± 1 °C, 75 ± 5% relative humidity, 12:12 hour light-dark cycle) by mass sib-mating and collective oviposition. Eggs were hatched in dechlorinated tap water and larvae were reared with a standard diet of fish food in 24 × 34 × 9 cm plastic trays at a density of about 200 larvae per tray. A maximum of 600 male and female adults were maintained in 30 × 30 × 30 cm screened cages with permanent access to a 10% sucrose solution. Adult females were fed on commercial rabbit blood (BCL, Boisset St Priest, France) through a membrane feeding system (Hemotek Ltd, Blackburn, UK) using pig intestine as the membrane. At each generation, eggs were collected and stored on dry pieces of paper towel for no longer than three months.

### Experimental infections

Mosquitoes were deprived of sucrose solution 24 hours prior to the infectious blood meal. Five to seven days after adult emergence, females were allowed to feed for 15 min from a membrane feeding system (Hemotek) that contained an artificial infectious blood meal maintained at 37 °C. The infectious blood meal consisted of two volumes of washed rabbit erythrocytes and one volume of viral suspension. Adenosine triphosphate was added to the blood meal as a phagostimulant at a final concentration of 10 mM. Rabbit erythrocytes were obtained from arterial blood collected the day before and washed 2 hours before the experimental infection. An aliquot of the artificial blood meal was collected immediately prior to blood feeding and stored at −80 °C for later titration by FFA as described above.

After virus exposure, fully engorged females were individually transferred to small (80-mm high and 44 mm in diameter) cardboard containers (Fig. 1) and maintained under controlled conditions (27 ± 1 °C, 75 ± 5% relative humidity, 12:12 hour light-dark cycle). Filter paper disks (blotting papers MN 218 B, Macherey-Nagel, Hoerdt, France) slightly larger in diameter than the cardboard containers were placed below each cardboard container. Small 1-cm^2^ filter paper squares were soaked in a colored sugar solution and placed on top of the cardboard containers. The colored sugar solution consisted of 5 g of Manuka Honey (Comvita UK Ltd, Slough, UK) diluted in 100 μL of blue food dye (Vahiné, Avignon, France,) and 10 mL of tap water.

### DENV RNA detection

DENV RNA was detected in mosquito organs (midgut, head, Malpighian tubules) freshly dissected from freeze-killed mosquitoes, and in mosquito excreta collected on filter paper. At each time point following virus exposure, mosquito excreta spots on the filter paper were excised using a scalpel and incubated for 20 min in 400 μL of PBS in a 2-mL microtube. After vortexing for 10 sec, 200 μL of solution were mixed with 300 μL of RNA lysis buffer. Total RNA from individual organs and excreta collections was extracted using the NucleoSpin RNA kit (Macherey-Nagel) according to the manufacturer’s instructions. At the final step, total RNA from each sample was eluted in 75 μL of RNase-free water.

Absolute quantification of DENV RNA was performed with a one-step reverse transcription quantitative polymerase chain reaction (RT-qPCR) assay. The RT-qPCR assay was modified from,^35^ to generate a 105-bp amplicon located in a conserved region of the DENV-1 *NS5* gene. The assay used the SuperScript III Platinum One-Step Quantitative Kit (Invitrogen) and was performed with 1x of reaction mix, 150 nM of PCR primers (forward: 5′-GGAAGGAGAAGGACTCCACA-3′; reverse: 5′-ATCCTTGTATCCCATCCGGCT-3′) and 0.5 μM of hydrolysis probe (5′-CTCAGAGACATATCAAAGATTCCAGGG-3′). Amplification was performed on a LightCycler 96 real-time thermocycler (Roche Diagnostics, Meylan, France) using the following program: RT at 53 °C for 20 min, polymerase activation at 95 °C for 2 min, followed by 45 PCR cycles of 15 sec at 95 °C and 40 sec at 56 °C.

For absolute quantification, a standard curve was generated in duplicate using 10-fold serial dilutions of total DENV RNA of known concentration. Compared to RNA standards produced by *in vitro* transcription from a cloned PCR amplicon, the use of total viral RNA for absolute quantification has the advantage of relying on more comparable conditions in template and standard reactions. Briefly, total RNA was isolated from KDH0026A virus stock using TRIzol (Life Technologies). Viral RNA concentration was determined using a different RT-qPCR assay targeting a 101-bp region of the DENV-1 envelope (*E*) gene. This RT-qPCR assay used a SYBR Green I kit (Roche Applied Science, Mannheim, Germany) and custom PCR primers (forward: 5′-TGCGCAAACTGTGCATTGAAG-3′; reverse: 5′-GTTCGCGTCTTGTTCTTC-3′). The target region of the *E* gene was previously amplified from KDH0026A and cloned into a pCR II-TOPO vector using the TOPO TA cloning kit (Invitrogen). The presence of the insert DNA was confirmed by PCR and Sanger sequencing. Plasmidic DNA was extracted using the miniprep kit (Qiagen). The plasmid was linearized by digestion with *SpeI* and the target sequence was transcribed *in vitro* using the T7 MEGAscript kit (Ambion, Austin, TX, USA) as recommended by the manufacturer. The *in vitro* transcribed RNA was treated with DNAse to digest the plasmid and purified using the NucleoSpin RNA XS kit (Macherey-Nagel). The RNA concentration was quantified with a NanoDrop spectrophotometer (NanoDrop Technologies Inc., Wilmington, DE, USA). RNA copy numbers were calculated based on the measured concentration and molecular weight of the RNA transcript.

All RT-qPCR assays included 10-fold serial dilutions of RNA standards ranging from 10^1^ to 10^7^ RNA copies/μL of reaction. The total number of DENV RNA copies present in each sample was calculated by multiplying the measured DENV RNA concentration by the elution volume (i.e., 75 μL) and the dilution factor.

Conventional detection of DENV RNA was performed with a two-step reverse transcription polymerase chain reaction (RT-PCR) assay. Total RNA was first reverse transcribed to complementary DNA (cDNA) with random hexamers using M-MLV Reverse Transcriptase (Life Technologies). The cDNA was amplified by 45 cycles of PCR using the set of primers targeting the *NS5* gene described above. Amplicons were visualized by electrophoresis on 2.5% agarose gels.

### Statistics

The tetrachoric function from of the R psych package^36^ was used to measure tetrachoric correlations between the presence or absence of DENV RNA between pairs of mosquito samples. The tetrachoric correlation is a technique to estimate the correlation between two theorized normally distributed continuous variables from two observed dichotomous variables^36^. Fisher exact tests were performed to examine the statistical significance of the correlations, with the absence of correlation between the presence of DENV RNA in excreta and in organs as the null hypothesis. *P*-values were adjusted for multiple testing by applying a Bonferroni correction.

A Cox regression model that included DENV blood meal titer as a covariate was used to compare viral dissemination and mosquito survival across virus doses. In the Cox regression model, the baseline hazard function is estimated non-parametrically and the times to events are not assumed to follow a specific statistical distribution^37^. Cox regressions were carried out with the R survival package^38^. A log likelihood test was used for model comparison. The assumption of proportional hazard for the virus dose covariate was verified by correlating the scaled Schoenfeld residuals with the Kaplan-Meier estimate of the survival function^39^.

All statistical analyses were performed in the R statistical environment^40^ and plotted with the R ggplot2 package^41^.

## Additional Information

**How to cite this article**: Fontaine, A. *et al*. Excretion of dengue virus RNA by *Aedes aegypti* allows non-destructive monitoring of viral dissemination in individual mosquitoes. *Sci. Rep*. **6**, 24885; doi: 10.1038/srep24885 (2016).

## Supplementary Material

Supplementary Information

## Figures and Tables

**Table 1:** Correlation between presence of DENV RNA in excreta and systemic viral dissemination.

	Excreta	Head	Malpighiantubules
Head	0.90***		
Malpighian tubules	0.87**	0.99***	
Midgut	0.70	0.80*	0.83**

The table shows tetrachoric correlation coefficients for the pairwise presence or absence of DENV RNA in the excreta, head, midgut and Malpighian tubules of *Ae. aegypti* females previously exposed to DENV. Females were exposed to several virus doses (10^7 ^FFU/mL, 4 × 10^5 ^FFU/mL or 10^5 ^FFU/mL) and collected at different time points (days 5, 8 and 15 post exposure) to cover a range of vector competence phenotypes. Viral dissemination is inferred from detection of DENV RNA in head tissues and/or Malpighian tubules. *P*-values represent the statistical significance of the correlations based on Fisher exact test and adjusted for multiple testing (*p < 0.05, **p < 0.01, ***p < 0.001).
