# Supplementary material for: Excretion of dengue virus RNA by Aedes aegypti allows non-destructive monitoring of viral dissemination in individual mosquitoes
Source: Sci Rep. 2016 Apr 27;6:24885. doi: 10.1038/srep24885 (PMC4846815; doi:10.1038/srep24885)
Supplement: Supplementary Information [file srep24885-s1.pdf]

Excretion of dengue virus RNA by *Aedes aegypti* allows non-destructive monitoring of viral dissemination in individual mosquitoes

Albin Fontaine[1,2,3]*, Davy Jiolle[1,3], Isabelle Moltini-Conclois[1,3], Sebastian Lequime[1,3,4], Louis Lambrechts[1,3]*

[1] *Insect-Virus Interactions Group, Department of Genomes and Genetics, Institut Pasteur, Paris, France*

[2] *Equipe Résidente de Recherche d'Infectiologie Tropicale, Division Expertise, Institut de Recherche Biomédicale des Armées, Brétigny-sur-Orge, France*

[3] *Centre National de la Recherche Scientifique, Unité de Recherche Associée 3012, Paris, France*

[4] *Université Pierre et Marie Curie, Cellule Pasteur UPMC, Paris, France*

* **Corresponding authors:** albinfont@gmail.com (AF); louis.lambrechts@pasteur.fr (LL).

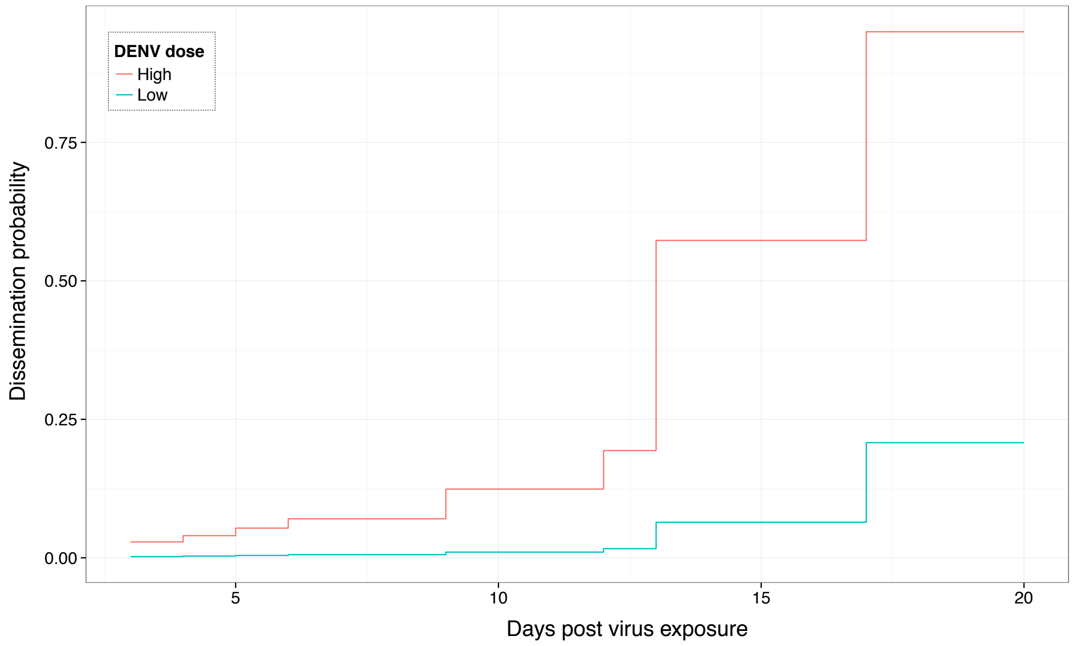

Supplementary figure 1: Kaplan-Meier plot of the probability of viral dissemination as a function of virus dose. Female mosquitoes were orally exposed to either 10^7 FFU/mL (high virus dose) or 4.5 x 10^5 FFU/mL (low virus dose) of DENV and scored daily for the presence of DENV RNA in their excreta over 27 days. Presence of DENV RNA in mosquito excreta was used as a proxy for systemic viral dissemination.
